# *Acinetobacter* Plasmids: Diversity and Development of Classification Strategies

**DOI:** 10.3389/fmicb.2020.588410

**Published:** 2020-11-13

**Authors:** Sofia Mindlin, Alexey Beletsky, Andrey Rakitin, Andrey Mardanov, Mayya Petrova

**Affiliations:** ^1^Institute of Molecular Genetics of National Research Centre “Kurchatov Institute”, Moscow, Russia; ^2^Institute of Bioengineering, Research Center of Biotechnology of the Russian Academy of Sciences, Moscow, Russia

**Keywords:** relaxases, replication initiator proteins, backbone and accessory regions, phylogenetic analysis, adaptation of bacteria

## Abstract

Bacteria of the genus *Acinetobacter*, with their numerous species common in various habitats, play a significant role as pathogens. Their ability to adapt to different living conditions is largely due to the presence of numerous plasmids containing the necessary adaptive genes. At the same time the diversity of *Acinetobacter* plasmids and their evolutionary dynamics have not been sufficiently studied. Here, we characterized 44 plasmids isolated from five permafrost *Acinetobacter lwoffii* strains, examined their relationship with plasmids of modern *Acinetobacter* strains and identified groups of related plasmids. For this purpose, we have developed a combined approach for classifying all known *Acinetobacter* plasmids. The classification took into account the size of plasmids, the presence and structure of the *rep* and *mob* genes, as well as the structure of their backbone and accessory regions. Based on the analysis, 19 major groups (lineages) of plasmids were identified, of which more than half were small plasmids. The plasmids of each group have common features of the organization of the backbone region with a DNA identity level of at least 80%. In addition, plasmids of the same group have similarities in the organization of accessory regions. We also described a number of plasmids with a unique structure. The presence of plasmids in clinical strains that are closely related to those of environmental permafrost strains provides evidence of the origin of the former from the latter.

## Introduction

A number of complete bacterial plasmid genomes has increased many times in recent years and continues to grow rapidly (Shintani et al., 2015; Lean and Yeo, 2017; Brovedan et al., 2019, 2020). Against this background, studies of their structure and functioning are noticeably lagging. In particular, not enough attention is paid to the study of the diversity of plasmids and their evolutionary dynamics in environmental bacterial populations.

Bacteria belonging to the genus *Acinetobacter* are a convenient model for such studies, since strains of different species of this genus are widespread and ubiquitous; they play an important role in various ecological niches, including soil, water, associations with various plants and animals, while many of them are human pathogens (Touchon et al., 2014). An important feature of *Acinetobacter* is also the presence of multiple plasmids in the same strain. In an attempt to study the diversity and prevalence of plasmids in different *Acinetobacter* species, various researchers tried to classify them using approaches based on a comparative analysis of the structure of replication initiator proteins (Bertini et al., 2010; Lean and Yeo, 2017; Cameranesi et al., 2017; Salto et al., 2018; Brovedan et al., 2020) or the structure of mobilization proteins (relaxases; Francia et al., 2004; Garcillán-Barcia et al., 2009; Smillie et al., 2010; Garcillán-Barcia and de la Cruz, 2013; Shintani et al., 2015; Brovedan et al., 2019).

It should be noted that both approaches have their limitations. Some plasmids do not contain known replication initiator proteins while others can contain not one but two or three replication genes or a recombinant replication gene (Bertini et al., 2010; Feng et al., 2016; Thomas et al., 2017; Salto et al., 2018; Salgado-Camargo et al., 2020), which prevents unambiguous identification of plasmids by this criterion. The system designed by Bertini et al. (2010) is rather limited for plasmids of other *Acinetobacter* species (Salto et al., 2018). Therefore, a universal classification system based on the analysis of sequences of replication initiator proteins does not currently exist. On practice, different groups of researchers use different systems to classify plasmids (Salto et al., 2018; Brovedan et al., 2019; Salgado-Camargo et al., 2020).

In comparison with replicases relaxases are less variable (Francia et al., 2004; Smillie et al., 2010; Salto et al., 2018) and their closely related variants are widespread among representatives of various species (Francia et al., 2004; Garcillán-Barcia et al., 2009). However, a number of plasmids do not contain the gene encoding relaxase (*mobA*) and it is necessary to use the analysis of the replication genes in this case.

Thus, one can conclude that there is not and cannot exist a universal system based on the analysis of only one plasmid protein, *rep, mob*, or another. Therefore, it is necessary to develop some new approaches for classification of plasmids.

We have sequenced the genomes of 44 plasmids isolated from environmental *Acinetobacter lwoffii* strains, which allowed to significantly expand the variety of known plasmids of this species. Related plasmids are also found among modern *Acinetobacter* strains. At the same time, an increasing number of strains belonging to different *Acinetobacter* species are isolated in the clinic, many of which contain plasmids with various sets of antibiotic resistance genes. Thus, a modern classification of *Acinetobacter* plasmids is needed for further research of the diversity and evolution of plasmids, as well as for diagnostic purposes.

In this study, we aimed to develop an improved classification system for *Acinetobacter* plasmids, starting from a detailed analysis of the plasmids of ancient *A. lwoffii* strains. By combining different methods for classification of plasmids of various sizes, we propose a “synthetic” approach to classify all known *Acinetobacter* plasmids and test its reliability in comparison with known approaches.

## Materials and Methods

### Bacterial Strains and Growth Conditions

The ancient *A. lwoffii* strains ED23-35, ED45-23, EK30A and VS15 and *A. pseudolwoffii* strain ED9-5a used in this study were previously isolated from 15 thousand to 3 million years old permafrost sediments collected from different regions of Kolyma Lowland (Petrova et al., 2002; Kholodii et al., 2004; Mindlin et al., 2016). The complete list of permafrost *A. lwoffii* plasmids is presented in Supplementary Table S1.

### Whole-Genome Sequencing and Assembly of Plasmids

Genomic DNA was isolated using the PowerSoil DNA isolation kit (Mo Bio Laboratories Inc., Carlsbad, CA, United States). The sequencing libraries for Illumina sequencing were prepared using the TruSeq nano DNA library prep kit (Illumina, United States) following the manufacturer’s instructions. The sequencing of this library on the Illumina MiSeq platform using Miseq Reagent Kit v3 (600 cycles). At least 90-fold sequence coverage was achieved for each genome. Paired overlapped reads were merged into longer reads using FLASH v1.2.11 (Magoč and Salzberg, 2011), and low quality read ends were trimmed using Sickle v.1.33 (option q = 30^1^). Genomic DNA was additionally sequenced on a MinION system (Oxford Nanopore, United Kingdom) using the 1D Genomic DNA by ligation protocol. These long reads were used to assembly the Illumina contigs into longer sequences. Hybrid assembly of Illumina and Nanopore reads was performed using Unicycler v. 0.4.8 (Wick et al., 2017). We identified circular contigs that contained genes for mobilization and/or replication of plasmids.

### Bioinformatics Analysis

For the assembly and analysis of plasmid genomes from ancient strains the program UGENE^2^ was used. Similarity searches were performed using BLAST on NCBI site (Altschul et al., 1997; Megablast for BlastN and blastp for BlastP) and the resulting alignments were checked manually. Open reading frames (ORFs) were searched using ORF Finder and BLAST software at NCBI. Conserved domains and motifs were identified using the NCBI conserved domain database (CDD; Marchler-Bauer et al., 2011) and the Pfam database (Finn et al., 2009). When annotating new plasmid genomes, we adhered to the recommendations developed by the authors of the review devoted to this problem (Thomas et al., 2017).

### Phylogenetic Analysis

For the phylogenetic analysis we analyzed genomes of all set of *Acinetobacter* plasmids from NCBI databases submitted before April 01, 2020. A total of 981 genomes were analyzed, including 44 plasmids from permafrost *A. lwoffii* (Supplementary Tables S1, S2). MobQ and RepB proteins were identified using PSI Blast search against pfam03389 and pfam01051 with *e*-value 1e-8 cutoff. Mob_H__EN_ and Mob_P_ proteins were identified using psiblast search with six iterations and *e*-value 1e-8 cutoff with Mob_HEN_ and Mob_P_ proteins lists from Garcillán-Barcia et al. (2009) as an input. As a result, *mob* genes were detected in 381 plasmids out of 981 analyzed. A Mob tree was inferred from the alignment of the first 300 amino acids of the N-terminal domain of relaxase proteins; Mob proteins shorter than 200 amino acids were discarded.

Proteins were clustered at 95% identity and 0.9 length reciprocal coverage threshold using blastclust. Proteins representing clusters were aligned using MUSCLE v3.8.31, the alignment was used as an input for maximum likelihood tree construction in PhyML v3.3 with default parameters. Two separate trees for Mob and Rep proteins were constructed and visualized using ggtree v2.2.1 R package.

### Classification and Identification of Plasmids Isolated From Permafrost and Modern *Acinetobacter* Strains

Mobilizable plasmids were classified based on the analysis of relaxases using BLAST. As reference sequences, sequences belonging to different families of relaxases described by the developers of the manuals were used (Francia et al., 2004; Garcillán-Barcia et al., 2009). The latest version of the classification system based on the analysis of the relaxases structure was also used (Salto et al., 2018). Non-mobilizable plasmids were classified based on the analysis of replication initiator proteins (Bertini et al., 2010). When forming groups (lineages) of closely related plasmids, the results of a comparative analysis of the structure of their backbone (basic) regions were used. Closely related plasmids from modern *Acinetobacter* strains were identified using BLASTn. Related plasmids from modern Proteobacteria strains were identified using the BLASTp program. Plasmids that not only had the same set of closely related genes in their backbone regions, but also had the same part of the accessory region adjacent to the backbone region, were assigned to the same group. The criteria for forming groups developed by us are described in more detail in the results.

## Results

### A Primary Classification of *Acinetobacter* Ancient Plasmids

**FIGURE 1:**
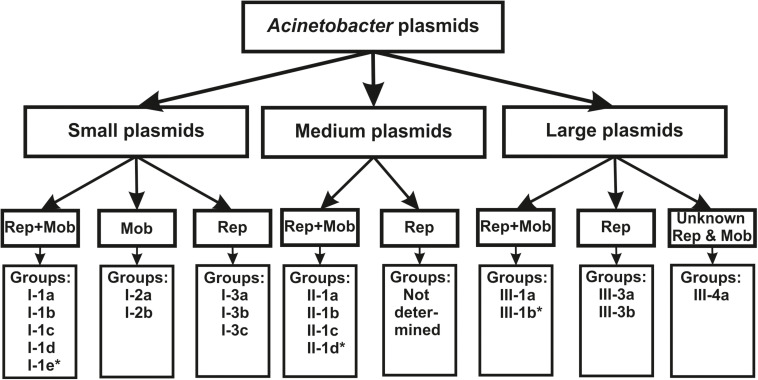
The classification scheme used in the work (see the text for details). ^∗^ plasmids from this group were found only in modern strains.

Sequencing of the complete genomes of five strains of the ancient *A. lwoffii* and *A. pseudolwoffii* strains produced complete circular sequences of 44 different plasmids present in these strains (Supplementary Table S1). Initially, we analyzed the molecular structure of these plasmids in order to group them by size and by the presence of genes encoding the *mobA* and *repB* proteins (Table 1 and Figure 1). All plasmids were divided into three categories, according to their sizes: large plasmids (more than 40 kb), medium-sized plasmids (12–40 kb) and small plasmids (2–12 kb). Each of these was marked with a Roman numeral depending on the size of the plasmids: I – small; II – medium, III – large. Then the plasmids of each category were divided into three parts, indicated by Arabic numerals: 1 – both mobilization and replication genes are present; 2 – only mobilization genes are present (this variant is found only in small plasmids); 3 – only replication genes are present (Table 1).

**TABLE 1:** A primary classification of plasmids from permafrost *Acinetobacter lwoffii* strains.

Large Plasmids (> 40 kb)		Medium Plasmids (12–40 kb)		Small plasmids (2–12 kb)		
Conjugative (III-1)	Non-conjugative (III-3)	*mobA-repB* (II-1)	*repB* (II-3)	*mobA-repB* (I-1)	*mobA* (I-2)	*repB* (I-3)
2	6	4	3	20	5	3
Total: 8 (18,6%)		Total: 7 (16,3%)		Total: 29 (65,9%)		
Conjugative: 2 (25%)		Mobilizable: 4 (57%)		Mobilizable: 26 (90%)		

Thus, based on the size and composition of the backbone region, we divided the ancient *Acinetobacter* plasmids into seven main blocks. It should be mentioned that small plasmids predominate in our collection and that most of them are mobilizable. Four of the seven medium- size plasmids also contained mobilization genes, while two of the eight large plasmids contained conjugative genes (Table 1). In total, mobilizable or conjugative plasmids predominate among plasmids originated from permafrost strains (70.5% of all plasmids).

### Groups (Lineages) of *Acinetobacter lwoffii* Plasmids Isolated From Permafrost

Further analysis revealed that within each block, distinct groups (lineages) of related plasmids can be distinguished. When identifying groups of related plasmids, several criteria were followed: (i) identification of mobilizable and conjugative plasmids was carried out by comparative analysis of the structure of their relaxases with those of known MobA/MobL families of plasmids; (ii) identification of non-mobilizable plasmids was carried out by comparative analysis of the structure of their replication initiator proteins with those of known groups of *Acinetobacter* plasmids; the presence of two or more replication genes in the same plasmid were taken into account; (iii) the size of the plasmids, as well as the presence and structure of the genes in their accessory region were also taken into account.

As a result, clearly delineated groups of plasmids have been found, which we designated in Latin letters (Table 2). Plasmids from a single group possess a common set of genes that make up the backbone region. Furthermore, the similarity of the nucleotide sequences of the backbone regions of representatives of each group is at least 80%. In addition, the plasmids of the same group often have similarities in the organization of the accessory region. Thus, all group I-1a plasmids contain *dif*-modules [a small mobile element surrounded by XerC/XerD recombination sites (Blackwell and Hall, 2017; Mindlin et al., 2019)] with different adaptive genes, while their position in the plasmid genome remain constant. Group I-1b plasmids contain very conserved mobilization genes, while replication genes are characterized by high variability. The plasmids of groups I-1c and I-1d, in addition to belonging to different families, differ in size (Supplementary Table S3). The plasmids of groups I-2a and I-2b are united by the absence of replication genes, while differ from each other in other parameters. In particular, their relaxases belong to different families, MOBQ and MOBHEN respectively. At the same time, group I-2a plasmids contain, in addition to the *mobA* gene, the *traD* gene encoding a binding protein (T4CP, coupling protein), which plays in conjugative plasmids a key role in the transfer of single-stranded DNA to a recipient cell (de Paz et al., 2010). As far as we know, the gene *traD* was not previously detected in small plasmids of the MOBQ family.

**TABLE 2:** Groups of plasmids revealed in studies of permafrost *Acinetobacter lwoffii* strains.

Group	Reference Plasmid [Accession No]	Structure of the Backbone Region	Family of Plasmids
**Small plasmids**			
I-1a	pALWED2.6 [CP032121.1]	*mobS-mobA/L-repB*	MOB_Q2_
I-1b	pALWED1.5 [CP032114.1]	*mobC-mobA/L-repB*	MOB_Q2_
I-1c	pALWEK1.3 [CP032106.1]	*mobS-mobA/L-repB*	MOB_Q2_
I-1d	pALWEK1.16 [MT675922]	*mobC-mobA-rep*	MOB_HEN_
I-2a	pALWED1.7 [CP032116.1]	*traD-mobA/L*	MOB_Q1_
I-2b	pALWED1.8 [LN873256.1]	*mobC-mobA/L*	MOB_HEN_
I-3a	pALWEK1.5 [KX426231.1]	*rep-repB*	REP_3 (AR3G8)
I-3b	pALWEK1.14 [MT675921]	*repA*	ND*
I-3c	pALWEK1.15 [MT675925]	*repB*	REP_3 (AR3G1)
**Medium plasmids**			
II-1a	pALWED3.2 [CP032287.1]	*mobA/L-repB*	MOB_Q1_
II-1b	pALWED3.5 [KX426230.1]	*mobA/L-repB*	MOB_Q1_
II-1c	pALWEK1.2 [CP032105]	*mobA/L-repB*	MOB_Q__2_
**Large plasmids**			
III-1a	pALWED2.2 [CP032117.1]	*trb*-operon-*parA-parB-tra-*operon	MOB_P_ (P11)
III-3a	pALWED1.2 [CP032112.1]	*parB-parA-repB*	REP_3 (AR3G4)
III-3b	pALWED3.1 [KX528687.1]	*repB-parA*	REP_3 (AR315)
III-4a	pALWED1.1 [KX426227.1]	*rep-parAB-tra-*operon	ND*

*ND, not determined.

Large non-conjugative plasmids were divided into two groups based on a comparative analysis of the structure of *repB* genes and the structure of accessory regions (Supplementary Table S3). All 5 plasmids of the first group contained a highly conserved backbone region with the replication initiator gene (*repB*) and genes for maintaining plasmid stability (*parA-parB*). At the same time, three plasmids of this group contained a single *repB* gene, while in the other two closely related plasmids (pALWVS1.1 and pALWEK1.1) we found a second *repB* gene with similarity to the *repB* gene of small mobilizable plasmids of group I-1c. Three additional genes related to the small I-1c plasmids, including the *mobA* gene, were found in these two plasmids next to the second *repB* gene, suggesting recombination between these two groups of plasmids. The structure of the plasmid pALWED3.1 was unique, and its replication initiator protein belonged to the AR3G15 group, whereas the Rep protein of the five above-described plasmids belonged to the AR3G1 group, according to classification proposed by Salto et al. (2018).

### Phylogenetic Analysis of Relaxases and Replication Initiator Proteins From *Acinetobacter* Plasmids and Their Comparative Analysis

Having found that permafrost plasmids can be divided into groups based on the homology of their nucleotide sequences, we decided to check whether the same groups can be distinguished based on phylogenetic analysis of their relaxases and replication initiator proteins. To this purpose, we constructed phylogenetic trees for the corresponding proteins.

We started this analysis by building a tree of Mob proteins found in permafrost plasmids. It occurred that the groups of plasmids presented in Table 2 were well separated on the basis of their *mob* gene sequences (data not shown).

**FIGURE 2:**
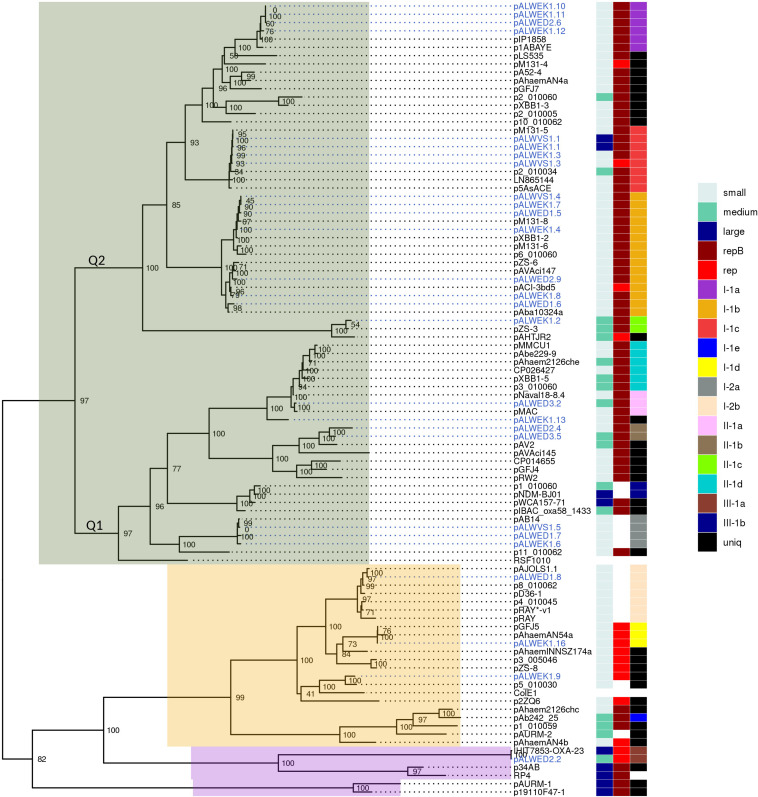
Phylogenetic tree of relaxases from *Acinetobacter* spp. plasmids. ML phylogenetic tree of MobA proteins of all *Acinetobacter* spp. plasmids from databases and from the collection analyzed in this study. Branch support values were calculated using approximate Bayes test. To the right of the tree, three columns show the characteristics of the plasmid encoding this relaxase: size (small, medium, or large), the presence or absence of a replication gene (*repB* or other), and affiliation to a group (with the exception of the reference plasmids RSF1010, ColE1, and RP4). Each of the plasmid groups (lineages) are marked in colors. Ancient plasmids, isolated from permafrost, are marked by blue. Relaxases belonging to the MOB_Q_ family are highlighted in gray, MOB_HEN_ – pink, and MOB_p_ – lilac.

We then tried to find out whether it is possible to identify new groups of plasmids in *Acinetobacter* strains by analyzing the phylogenetic tree of relaxases. For this purpose, a new tree was built (Figure 2), significantly expanding the previous one by including relaxases of modern *Acinetobacter* strains available in the database (see section “Materials and Methods”). The tree topology generally reflected the phylogenetic relationships of Mob proteins from different families (Garcillán-Barcia et al., 2009). Relaxases of most groups belonged to the MOB_Q_ family, of two groups – to the MOB_HEN_ family, and one group of large conjugative plasmids contained relaxase from the MOB_P_ family. As can be seen from Figure 2, the majority of modern plasmids belongs to one of the groups revealed among the ancient plasmids. We have found only three groups (I-1e, II-1d, and III-1b), whose members were not found among the ancient plasmids. In these groups the similarity of relaxases also reflected the relationship of the plasmids bearing them.

Recently, Salgado-Camargo et al. (2020) showed that the number of groups (lineages) of plasmids common among strains of *A. baumannii* is 21, with the total number of analyzed plasmids exceeding 170. Thus, the variety of *A. baumannii* plasmids is limited and is apparently associated with their ability to increase the adaptive properties of host bacteria. In our work, we have shown the validity of this conclusion on a much more extensive material, since about 980 plasmids of strains belonging to different species of *Acinetobacter* were analyzed. In total, we identified 19 groups (lineages) of *Acinetobacter* plasmids. Their true number is undoubtedly greater, given the limited use in our work of analysis of plasmid groups not bearing relaxase genes. At the same time, we demonstrated a wide distribution of plasmids from some lineages among various *Acinetobacter* species and even among representatives of other genera (group III-1a).

However, some plasmids are unique and only in some cases have a similarity of less than 80% with representatives of the closest group. Among the ancient plasmids of *A. lwoffii*, we found five such unique plasmids, pALWEK1.9, pALWEK1.13, pALWED1.3, pALWED2.3, and pALWVS1.2 (Supplementary Table S3). Perhaps such plasmids have a narrow host range and are spread only among environmental strains and therefore are poorly represented in the database.

**FIGURE 3:**
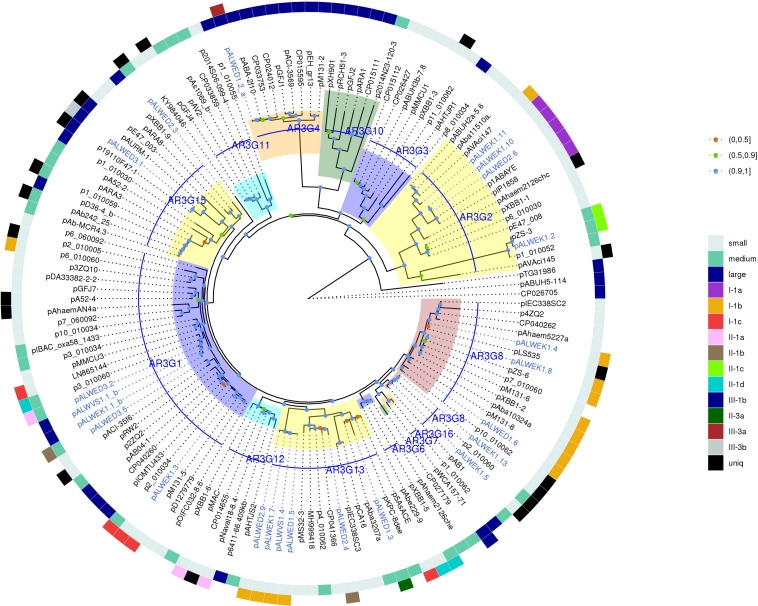
Phylogenetic tree of Rep_3 domain replication initiator proteins from *Acinetobacter* spp. plasmids. ML phylogenetic tree of Rep_3 (Pfam01051) proteins of all *Acinetobacter* spp. plasmids from databases and from the collections analyzed in this study. Proteins were clustered at 95% identity, only a selection of genes from each cluster was used for tree construction. Branch support values were calculated using approximate Bayes test. The characteristics of plasmid encoding this RepB are shown in the two outer circles: size (small, medium, or large), and affiliation to a group. Each of the plasmid groups (lineages) are marked in colors. Ancient plasmids, isolated from permafrost, are marked by blue. Branches corresponding to the different AR3G groups are highlighted in different colors. Bootstrap values are split into three intervals, shown as colored circles: [100, 90] as blue, [90, 50] as green, and [50, 0] as orange.

Next, we constructed a phylogenetic tree for plasmids containing *rep* genes, including plasmids carrying both *rep* genes and *mob* genes (Figure 3). As can be seen, the topology of the resulting tree coincides with that of the previously published phylogenetic analysis (Salto et al., 2018; Walter et al., 2020). In particular, all previously described AR3G groups are clearly represented (Figure 3). At the same time, plasmids with similar relaxases and very similar structure may encode Rep proteins belonging to different AR3G groups (see lines on the map shown in different colors). Therefore, one can conclude that there is a lack of coherent evolution of genes encoding proteins involved in the mobilization and replication of plasmids. Moreover, the phylogenetic proximity of mobilization proteins reflects the similarity of the plasmids carrying them, while in the case of replication initiator proteins, such a relationship is often absent.

However, permafrost plasmids without *mob* genes but having related *rep* genes often have a similar structure, i.e., they clearly belong to the same group. Consequently, at least in some cases, *rep* genes can be used to classify plasmids in the absence of *mob* genes. But it should be borne in mind that one clade of Rep can include proteins encoded by non-related plasmids characterized by different structure. Some of these plasmids contain two or even three different *repB* genes. The findings suggest that plasmids are able to exchange by replication genes. For this reason, when classifying plasmids containing *mob*, it is necessary to rely on the analysis of relaxases.

### Identification of Modern *Acinetobacter* Plasmids Closely Related to Ancient Plasmids of Different Groups

In parallel with the construction of phylogenetic trees for relaxases and replicases we performed the search of plasmids from modern strains closely related to such from permafrost *A. lwoffii* strains. Preliminarily, from each group a single plasmid was selected and used as a reference plasmid when searching relatives among modern plasmids (Table 2). In most cases plasmids of the same groups that were found among permafrost strains of *A. lwoffii* were common among modern *Acinetobacter* strains (Supplementary Table S3). The exception was predominantly medium-sized plasmids, especially those that did not contain the *mobA* gene. Perhaps this limits their transfer into the cells of other strains thus explaining the narrow host range of these plasmids. More complex patterns occur in a comparative analysis of permafrost and modern large plasmids. In the case of conjugative plasmids pALWED2.2 (group III-1a) and pALWED1.1 (group III-4a) closely related modern plasmids were discovered by detailed comparative analysis of their structure with that of permafrost plasmids. Plasmids closely related to pALWED2.2 carrying the same conjugative transfer system (Ewers et al., 2016; Silva et al., 2018) contain antibiotic resistance genes (*bla*_oxa23_ and *strA-strB*) in their accessory region. Modern derivatives of pALWED1.1 were broadly spread among strains of different *Acinetobacter* species but have not been previously identified as conjugative plasmids (see more detailed information presented below).

In the case of non-conjugative plasmids, when identifying related modern plasmids in addition to the comparative analysis of *repB* genes, the structure of *parA* and *parB* genes encoding a plasmid partition system was also taken into account. As a result, it was possible to detect closely related plasmids for plasmids of group III-3a as well as for plasmid pALWED3.1 (group III-3b; Supplementary Table S3).

Importantly, the list of modern plasmids contains plasmids originated from strains belonging to different *Acinetobacter* species and isolated from a variety of sources, suggesting horizontal transfer of the most successful (adapted) plasmid variants (Supplementary Tables S3, S4).

Data demonstrating the spread of plasmids of group 1-2a among ancient and modern strains of *Acinetobacter*, typical for other groups of plasmids, are presented in Table 3. It can be seen that closely related plasmids belonging to the same group are found among the strains of various *Acinetobacter* species inhibiting different ecosystems. It is significant to note that some strains were isolated from environment, while others are clinical strains or sewage dwellers.

**TABLE 3:** Distribution of plasmids belonging to group I-2a among different *Acinetobacter* strains.

Source	*Acinetobacter* Species: Strains	Source
Permafrost	*A.lwoffii:* ED23-35, EK30A	Kolyma, Russia
	*A. pseudolwoffii:* ED9-5a	Kolyma, Russia
Soil	*A. baumannii:* DS002	India
	*A. soli:* GFJ2	Thailand
Gold mine	*A. lwoffii:* ZS207	Zloty Stok, Poland
Sewage	*A. wuhouensis:* WCHA60, WCHA62	China
	*A. chinensis:* WCHAc010005	China
	*A.baumannii:*VB16141	India
Homo sapiens	*A. baumannii:* VB16141, A85	India, Australia
	*A. pittii*: C54	Australia

### The Novel *Acinetobacter* Plasmids

Several groups of plasmid identified in the present work were analyzed in more detail. In particular, plasmids of three groups, one containing large plasmids (III-4a) and two containing small plasmids (I-1a; I-2a) are widely spread among modern *Acinetobacter* strains but have not been described previously.

#### Group III-4a

Among large plasmids, the most interesting is the extensive group III-4a of conjugative plasmids, since members of the new group differ from previously described conjugative plasmids by the structure of the minimal replicon and the absence of known relaxase and replicase genes. Plasmids from the group III-4a were isolated from strains of various *Acinetobacter* species. We found their prototype plasmid pALWED1.1 among the plasmids of our collection of ancient strains and, based on preliminary studies, revealed its close relationship with plasmids of modern strains. It should be noted that among the modern large conjugative plasmids, there are other variants that are not related to pALWED1.1, but that also do not have known relaxases and replicases (for example, pA297-3 described by Hamidian et al., 2016 and pNDM-BJ01 and related plasmids described by Hu et al., 2012). Perhaps, for such plasmids, the classification can be based on the structure of the *tra* operon.

#### The Group I-2a of MOB_Q_ Family

Among the small plasmids, the most interesting are the plasmids of the two groups belonging to MOB_Q_ family. Group I-2a is the group of the smallest (4–8 kb) plasmids of the MOB_Q_ family characterized by several distinctive properties: (i) by the absence of the gene *repB*; (ii) by the unique structure of the gene *mobA* which contains only one domain corresponding to the relaxase region of *mobA* from RSF1010; (iii) by the absence of the gene *mobC*; (iv) by the presence of the *traD* gene encoding the coupling conjugational protein located in the plasmid backbone region.

**FIGURE 4:**
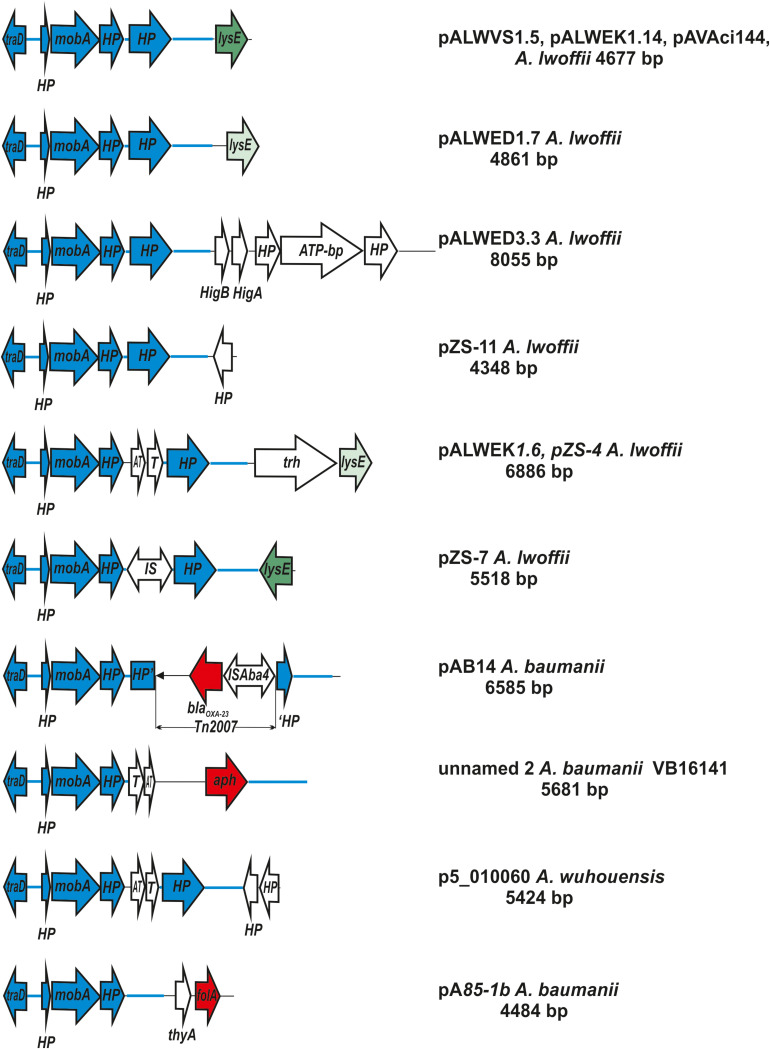
Comparative genetic structures of plasmids belonging to the group I-2a. The locations and polarities of genes and ORFs are shown by arrows; genes and ORFs of the backbone regions – by blue arrows. LysE gene is indicated by green arrow, different shades in different variants. Antibiotic resistance genes: *aph* (resistance to aminoglycosides), *bla*_oxa23_ (resistance to beta-lactams) and *folA* (trimethoprim resistance) are depicted as a red arrow. The rest genes and ORFs of accessory region are indicated as white arrows. *trh*, gene encoding tryptophan 7-halogenase. Designations of gene products are as follows: HP, hypothetical protein; ATP, ATP-binding protein; HigB, T, toxin; HigA, AT, antitoxin. The picture is drawn to scale.

Plasmids of this group are extremely widespread among *Acinetobacter* strains, belonging to different species of this genus (Supplementary Tables S4, S5 and Figure 4). Some strains contain two (or even three) such plasmids. In particular, we found three strains (*A. lwoffii* EK30A, *A. lwoffii* M2a, and *A. schindleri* HZE33-1) that each contained two plasmids of this group; one strain of *A. lwoffii* ZS207 isolated from Zloty Stok gold mine in Poland contained three plasmids (pZS-4, pZS-7, pZS-9) carrying genes *mobA* and *traD* and belonging to group I-2a.

It should also be noted that the same plasmid, with only a few nucleotide substitutions, could be detected in different *Acinetobacter* strains. So, virtually the same plasmid pALWEK1.6 was found in three different *A. lwoffii* strains: permafrost strain EK30A, the above mentioned strain ZS207 and a strain M2a isolated from a sample of honey in Hungary (Veress et al., 2020). Plasmid pALWVS1.5 was found in two different strains of *A. lwoffii* (VS15 and EK30A) isolated from different permafrost samples at different times. Finally, we found plasmid pVB2486_4 in three different strains of *A. baumannii*. Most likely, that the wide distribution of plasmids belonging to I-2a group can be explained by the presence in their genome of a pair of genes *mobA-traD* that ensure their effective mobilization. A possible effect of these plasmids on bacterial adaptability cannot also be excluded.

Interestingly, we found in the GenBank (whole-genome shotgun contigs) a strain identified by the authors as *Prolinoborus fasciculus* CIP 103579T (family Neisseriaceae) which contained a plasmid almost identical to the reference plasmid pALWED1.7. It seems to us, however, that this fact itself needs a confirmation.

It should be mentioned that accessory regions of different plasmids differed from each other (Supplementary Table S4 and Figure 4). Most of them contain genes that are thought to possess various adaptive functions. Two plasmids isolated from clinical strains of *A. baumannii* contained antibiotic resistance genes (resistance to beta-lactams determined by transposon Tn*2007* and resistance to aminoglycosides). Plasmids isolated from samples of permafrost, antimony deposits, soils, water and honey as well as sewage, contained genes for the metabolism of various amino acids. In some plasmids, accessory genes have not been identified (Supplementary Table S4).

#### The Group I-1a of MOB_Q_ Family Plasmids

**FIGURE 5:**
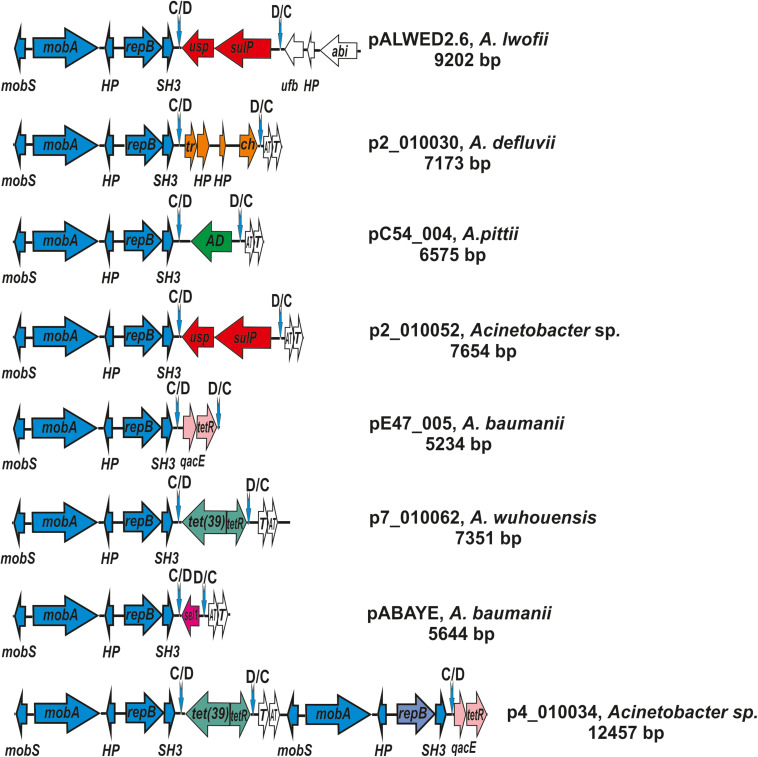
Comparative genetic structures of plasmids belonging to the group I-1a. The locations and polarities of genes and ORFs are shown by arrows; genes and ORFs of the backbone regions – by blue arrows; the components of the *dif* modules – by arrows with different colors; genes and ORFs of accessory regions – by white. Vertical arrows indicate p*dif* sites with the orientation of the subsites shown above. Designations of genes present in *dif* modules are as follows: *usp*, universal stress protein; *sulP*, sulfate permease; *tet*(39), tetracycline resistance; *tet*R, repressor; *sel1*, Sel1-repeat protein. Designations of gene products are as follows: HP, hypothetical protein; Tr, XRE family transcriptional regulator; *ch*, hydrolase; AD, zinc-dependent alcohol dehydrogenase family protein; *qacE*, QacE family quaternary ammonium compound efflux SMR transporter; T, toxin; AT, antitoxin; *tetR*, TetR/AcrR family transcriptional regulator. The picture is drawn to scale.

The prototype of this group is the plasmid pALWED2.6 (9,202); CP032121.1) isolated from the permafrost *A. lwoffii* strain ED45-23. The backbone region of pALWED2.6 contains mobilization genes *mobS* and *mobA* as well as the replication initiator gene *repB* and two additional ORFs encoding an SH3 domain protein and an unidentified protein. This region is present in all multiple derivatives of pALWED2.6 which form the I-1a group of plasmids including ancient as well as modern plasmids (Figure 5). It should be mentioned that the whole backbone region with both *mobA* and *repB* genes varies only slightly in different plasmids of this group. It can be assumed that unlike to other groups of plasmids, the relaxase and replicase genes of these plasmids evolved together.

The second distinctive feature of the I-1a group of plasmids is the presence of a *dif* module adjacent to the backbone region. While the location of the *dif* modules in different plasmids is the same, these are different modules containing different structural genes (Figure 5). In particular, the *dif* module of the plasmid pALWED2.6 contains the *sulP* gene encoding the sulfate permease and the *uspA* gene encoding a universal stress protein. Plasmid p1ABAYE from *A. baumannii* strain contains a *sel1* module encoding a putative virulence factor, and plasmid p7_010062 from *A. wuhouensis* carries a *tet39* module with *tetA39-tetR* genes encoding tetracycline resistance. *Dif*-modules of the remaining plasmids carry some other adaptive genes (Figure 5).

It should also be noted that in addition to the small plasmids characteristic of group I-1a, we found three medium-sized plasmids constructed from repeating elements (Figure 5).

Two such plasmids (p4_010034; p4_010060) contained backbone regions and two different *dif*-modules each (Figure 5). The backbone regions carried the *mobS* and *mobA* mobilization genes, the gene *repB* encoding the replication initiator protein and two additional genes encoding the SH3 domain protein and the hypothetical protein (Figure 5). Interestingly, the first of the backbone regions was almost identical to the corresponding region of small plasmids, while the second region contained identical mobilization genes, but different *repB* genes (77% identity level). The genes encoding toxin-antitoxin system were represented in one copy (Figure 5). The largest third plasmid pAcsw19-3 (38391 bp; CP043310.1), found in *A. johnsonii* Acsw19, contained five almost identical copies of the small plasmid, closely related to plasmid p2_010030 [CP029390.2] isolated from *A. defluvii* strain WCHA30. Accordingly, it contained five almost identical copies of the backbone regions, five copies of the genes encoding toxin-antitoxin system, and five *dif*-modules with a gene encoding cysteine hydrolase. The order of the genes in each copy was the same.

Plasmid pIP1858 (KP890934.1) from *A. baumannii* BM2686 strain differs from all other plasmids of the group I-1a. Whereas its backbone region is typical for the group, the accessory region, instead of the *dif* module, contains a complex structure consisting of two different IS elements surrounding three structural genes including an aminoglycoside resistance gene (Yoon et al., 2016). Most likely, the plasmid pIP1858 arose from a typical plasmid of group I-1a as a result of some recombination event(s).

The mechanism that ensures the replacement of *dif* modules located in the plasmids remains unknown. We determined the comparative structure of the plasmid *dif* sites (p*dif*) surrounding various *dif* modules, and found that most of the modern variants differ from the variant found in the ancient plasmid. The greatest variability is found in the central region of the p*dif* sites (dif^cent^; Supplementary Figure S1). It can be assumed that the replacement of *dif* modules can be provided by the site-specific recombination system XerC/XerD (Bloor and Cranenburgh, 2006; Carnoy and Roten, 2009; Castillo et al., 2017; Cameranesi et al., 2018) and may occur in two stages: (1) the old module is excluded from the plasmid, leaving a recombinant p*dif* site in it; (2) a new module is integrated into the plasmid by recombination with the recombinant p*dif* site. A possible alternative mechanism is a recombination through dif sites (Cameranesi et al., 2018).

## Discussion

In the present work, we have used a large collection of plasmids from permafrost strains of *A. lwoffii* to analyze the diversity of plasmids of various *Acinetobacter* species and to classify them. The main emphasis was on the analysis of small mobilizable plasmids, based on the structure of the *mobA* genes encoding relaxases. The structure of other genes of the backbone and accessory regions was also taken into account. It was found that: (i) a limited number of plasmid groups are distributed both in the environment and in the clinic; (ii) plasmids of modern *Acinetobacter* strains have their homologs among ancient strains, which allows us to consider the latter as the progenitors of the former; (iii) in the MOB_Q_ family, new groups of plasmids were identified and characterized; (iv) a novel group of large putative conjugative plasmids was discovered.

Thus, we have confirmed and expanded the conclusions of other authors about the prospects of using a classification system based on the analysis of the structure of relaxases in mobilizable and conjugative plasmids (Francia et al., 2004; Garcillán-Barcia et al., 2009; Garcillán-Barcia and de la Cruz, 2013; Shintani et al., 2015). However, an attempt to use for classification of non-mobilizable plasmids the analysis of the structure of replication initiator proteins was not that successful. In particular, when comparing phylogenetic trees constructed using either MobA or RepB proteins their mismatch was revealed, indicating not coherent evolutionary variability of the *mobA* and *repB* genes (Figures 2, 3). In a number of cases, we also found that plasmids carrying related *repB* genes can be completely different in structure (Supplementary Table S3). Furthermore, in two of the eight large plasmids studied, we found two different *repB* genes. Therefore, in the absence of relaxase genes on the plasmid, we recommend to take into account the structure of other genes of the backbone and accessory regions for classification.

In a recently published work (Salgado-Camargo et al., 2020), the authors classified plasmids of only one species, *A. baumannii*, based on the similarity of their Rep proteins and additional regions, excluding Mob proteins. Even in such a limited sample, they were faced with the fact that two groups of similar plasmids may contain different *rep* genes. As can be seen from our study for the entire genus *Acinetobacter*, the classification of plasmids based only on the analysis of replication initiator proteins is associated with significant inaccuracies.

The general structure of the members of each group of plasmids can remain unchanged for thousands of years. At the same time, comparative analysis of the structure of permafrost and modern plasmids from the same group allowed to identify changes in the structure of plasmids with changing living conditions, which confirmed the results of previous studies (Vallenet et al., 2008; Gillings et al., 2008; Brovedan et al., 2019). We showed that the main changes occur in an accessory region of plasmids and do not usually affect the structure of the backbone region. Moreover, in the plasmids of clinical strains, even small ones, we often revealed insertions of antibiotic resistance and virulence genes. Thus, along with medium-size and large plasmids, small plasmids of *Acinetobacter* likely contribute to the acquisition of properties necessary for the adaptation of host bacteria to changing living conditions.

## Data Availability Statement

The datasets generated in this study can be found in online repositories. The names of the repository/repositories and accession number(s) can be found below: https://www.ncbi.nlm.nih.gov/genbank/, KX426229.1 CP032117.1 CP032118.1 CP032119.1 CP032120.1 CP032121.1 CP032122.1 CP032123.1 CP032124.1, KX528687.1 CP032287.1 CP032288.1 KX426230.1 CP032290.1 MT675918, KX426227.1 CP032112.1 KX426228.1 CP032113.1 CP032114.1 CP032115.1 CP032116.1 LN873256.1, KX426232.1 MT675923 MT675924 MT319099 MT675926, CP032102.1 CP032105.1 CP032106.1 CP032107.1 KX426231.1 CP032108.1 CP032109.1 CP032110.1 CP032111.1 CP032103.1 CP032104.1 MT675919 MT675920 MT675921 MT675925 MT675922.

## Author Contributions

MP had the initial idea, which was developed into a project together with SM. AM, AB, AR, and MP conducted the sequencing, assembly of plasmids and genome annotation. SM and MP designed the tables. AB and MP designed the figures. MP, AB, and SM made the analysis of MobA proteins. AB and MP analyzed the RepB proteins. SM, MP, and AM wrote the manuscript. All authors performed the bioinformatic analysis and contributed to manuscript revision, and approved the submitted version.

## Conflict of Interest

The authors declare that the research was conducted in the absence of any commercial or financial relationships that could be construed as a potential conflict of interest.

## Footnotes

1 https://github.com/najoshi/sickle

2 http://unipro.ru/

## Supplementary Material

Supplementary Figure 1 Alignment of the recombination *dif* sites flanking *dif* modules in plasmids of group I-1a.

Supplementary Table 1 Plasmids from permafrost *Acinetobacter lwoffii* strains.

Supplementary Table 2 Analyzed *Acinetrobacter* plasmids.

Supplementary Table 3 Groups of *Acinetobacter* plasmids.

Supplementary Table 4 Small *Acinetobacter* plasmids belonging to the Group I-2a of MOBQ family.

Supplementary Table 5 Distribution of MOB_Q_ plasmids from I-2a group among modern *Acinetobacter* strains (whole genome shotgun contigs).
