# Supplementary figures and images for: Acinetobacter Plasmids: Diversity and Development of Classification Strategies
Source: Front Microbiol. 2020 Nov 13;11:588410. doi: 10.3389/fmicb.2020.588410 (PMC7693717; doi:10.3389/fmicb.2020.588410)

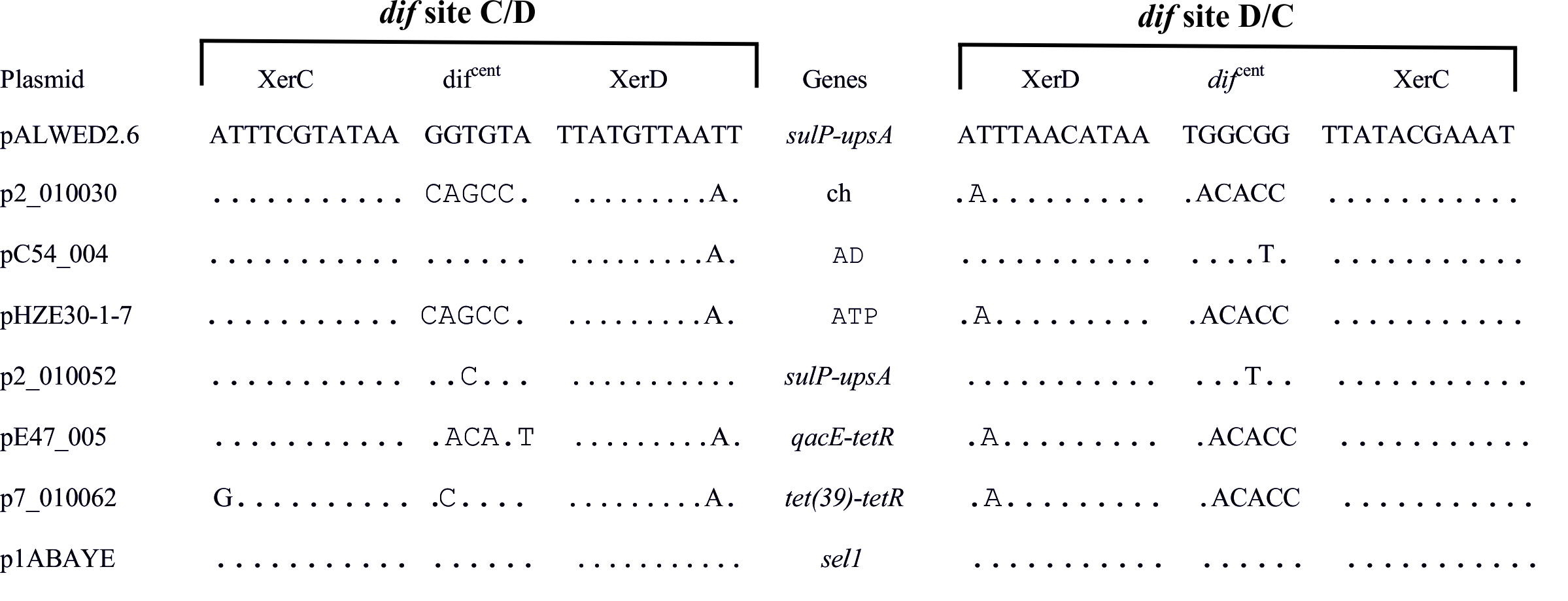

Supplement: Supplementary Figure 1 — Alignment of the recombination dif sites flanking dif modules in plasmids of group I-1a. [file Image_1.JPEG]
