# Supplementary material for: Acinetobacter Plasmids: Diversity and Development of Classification Strategies
Source: Front Microbiol. 2020 Nov 13;11:588410. doi: 10.3389/fmicb.2020.588410 (PMC7693717; doi:10.3389/fmicb.2020.588410)
Supplement: Supplementary Table 1 — Plasmids from permafrost Acinetobacter lwoffii strains. [file Table_1.doc]

**Table S1. Plasmids from permafrost *A.lwoffii* strains**

| **Strain** | **Plasmid** | **Length, bp** | **Accession no** |
| --- | --- | --- | --- |
| ED23-35 | pALWED1.1  pALWED1.2  pALWED1.3  pALWED1.4  pALWED1.5  pALWED1.6  pALWED1.7  pALWED1.8* | 287,630  47,739  16,071  14,118  6,711  5,596  4,61  4,135 | KX426227.1  CP032112.1  KX426228.1  CP032113.1  CP032114.1  CP032115.1  CP032116.1  LN873256.1 |
| ED45-23 | pALWED2.1  pALWED2.2  pALWED2.3  pALWED2.4  pALWED2.5  pALWED2.6  pALWED2.7  pALWED2.8  pALWED2.9 | 191,611  43,259  22,771  10,584  11,416  9,202  8,811  8,120  6,321 | KX426229.1  CP032117.1  CP032118.1  CP032119.1  CP032120.1  CP032121.1  CP032122.1  CP032123.1  CP032124.1 |
| ED9-5A | pALWED3.1  pALWED3.2  pALWED3.3  pALWED3.5  pALWED3.6  pALWED3.7 | 138,028  15,657  8,055  16,568  185,734  9,958 | KX528687.1  CP032287.1  CP032288.1  KX426230.1  CP032290.1  MT675918 |
| VS15 | pALWVS1.1  pALWVS1.2  pALWVS1.3  pALWVS1.4  pALWVS1.5** | 134,767  15,780  10,985  11,964  4,677 | KX426232.1  MT675923  MT675924  MT319099  MT675926 |
| EK30A | pALWEK1.1  pALWEK1.2  pALWEK1.3  pALWEK1.4  pALWEK1.5  pALWEK1.6  pALWEK1.7  pALWEK1.8  pALWEK1.9  pALWEK1.10  pALWEK1.11  pALWEK1.12  pALWEK1.13  pALWEK1.14  pALWEK1.15  pALWEK1.16 | 209,982  12,173  10,346  8,635  8,227  6,886  6,691  5,323  5,223  9,203  8,399  11,382  8,910  4,760  2,621  4,130 | CP032102.1  CP032105.1  CP032106.1  CP032107.1  KX 426231.1  CP032108.1  CP032109.1  CP032110.1  CP032111.1  CP032103.1  CP032104.1  MT675919  MT675920  MT675921  MT675925  MT675922 |

* pALWED1.8 (Kurakov et al, 2016) also present in strains VS15 и EK30A
