# Supplementary material for: Acinetobacter Plasmids: Diversity and Development of Classification Strategies
Source: Front Microbiol. 2020 Nov 13;11:588410. doi: 10.3389/fmicb.2020.588410 (PMC7693717; doi:10.3389/fmicb.2020.588410)
Supplement: Supplementary Table 3 — Groups of Acinetobacter plasmids. [file Table_3.doc]

**Table S3. Groups of *Acinetobacter* plasmids**

| Group | Structure of the  backbone region | Family of plasmids | Related permafrost plasmids | Related  modern plasmids | | Comments | |
| --- | --- | --- | --- | --- | --- | --- | --- |
| Small plasmids | | | | | | | |
| I-1a | *mobS-mobA/L-repB* | MOB_Q2_ | pALWED2.6 pALWEK1.10; pALWEK1.11; pALWEK1.12 | *Acinetobacter* sp; p2_010052 (7,6); *A. baumannii*; p1ABAYE (5,6);*A. wuhouensis*; p7_010062 (7,3); *A.defluvii* p2_010030 (7,2) | | The accessory region of all plasmids contains different *dif* modules in the same position | |
| I-1b | *mobC-mobA/L-repB* | MOB_Q2_ | pALWED1.5; pALWED1.6; pALWED2.8; pALWED2.9; pALWVS1.3; pALWEK1.4; pALWEK1.7; pALWEK1.8 | *A.wuhouensis* p2_010062 (28,4) *A. baumannii* p3ABSDF (24,9) | | The identity level of the *repB* gene varies from 79% (in part of the gene) to 100% regardless of changes in the *mobA /mobL* gene | |
| I-1c | *mobS-mobA/L-repB* | MOB_Q2_ | pALWEK1.3; pALWED1.4; pALWED2.5; pALWED2.7; pALWVS1.4; pALW*VS1.1;* pALW*EK1.1* | *A.baumannii*; pS30-1 (18,2); *Acinetobacter s*p. (7,6); *A. baumannii* pTS11291 (11,3); *A. schindleri* p6AsACE (7,9); *A. radioresistens* DSM 6976, pARA5 (9,4) | | pALWVS1.1 and pALWEK1.1 are large plasmids containing the additional *mobS-mobA/L-repB* region inherited from the small plasmid | |
| I-1d | *mobC-mobA-rep* | MOB_HEN_ | pALWEK1.16 | *A.haemolyticus* pAhaemAN54a (4,7); *A.soli* pGFJ5 (5,1) | | The trans-esterase (relaxase) domain of MobA proteins are almost identical in pALWEK1.16 and modern plasmids while the MobA second domains (helicase) are very different | |
| I-1e | *repB-parA- mobC-mobA* | MOB_HEN_ | - | *A. baumannii* pAb242_25 (24,8), pAb825_36 (35,7), *A. seifertii* pAs1069_a (24,7) | | Plasmids from this group were found only in modern strains | |
| unique | *mobC-mobA -repA* | MOB_HEN_ | pALWEK1.9 | *-* | | - | |
| unique | *mobA/L-repB* | MOB_Q1_ | pALWEK1.13 | Only distant similarity (72% with partial coverage). *A.haemolyticus*, pAhaem11616e (10,7); *A.schindleri*, pHZE23-1 (6,3) | |  | |
| I-2a | *traD-mobA/L* | MOB_Q1_ | pALWED1.7; pALWED3.3; pALWVS1.5; pALWEK1.6 | *A.wuhouensis*, p5_010060 (5,4); *A.baumannii*, №2 (5,7); *A. soli*, pGFJ6 (4,066); *A. pittii*, pC54_005 (4,5); *Prolinoborus fasciculus* CIP 103579T, contig_163 (4,9) | | Some modern plasmids contain antibiotic resistance genes | |
| I-2b | *mobC-mobA/L* | MOB_HEN_ | pALWED1.8 (found in ED23-35; VS15; EK30A) | *A. johnsonii*, pAJOLS1.1 (4,1); *A.wuhouensis*, p8_010062 (5,6)*; A.baumannii*, pD36-1 (4754)*A.baumannii*, pRAY* (6,1) | | Modern plasmids (except pAJOLS1.1) do not contain the *aadA27* gene | |
| I-3a | *rep-repB* | REP_3 (AR3G8) | pALWEK1.5 | *A.baumannii* CAM180-1, pCAM180B (16,1); *A.cumulans* WCHAc060092, p2_060092 (18,8); *A.wuhouensis* WCHA60, p2_010060 (30,8); *Acinetobacter* sp. WCHAc010034, p6_010034 (10,9 kb); *A. johnsonii* IC001, pIC001C (6,9) | |  | |
| I-3b | *repB* | ND* | pALWEK1.14 | *Acinetobacter s*p. WCHA45 p1_010045 (7,5) | |  | |
| I-3c | *repB* | REP_3 (AR3G1) | pALWEK1.15 | *Acinetobacter s*p. WCHA4, p1_010045 (7,5) | |  | |
| Medium plasmids | | | | | | | |
| II-1a | *mobA/L* | MOB_Q1_ | pALWED3.2 | *A. baumannii*, pMAC (9540); *A.lwoffii* M2a; pAVAci94 (16,9): *Acinetobacter* sp. NEB149, pMsp22 (22,0*); A. haemolyticus* 2126ch, pAhaem2126chd (10,6); *A. pittii* WCHAP005046, p2_005046 (10,5); *A.nosocomialis* SSA3, pSSA3_1 (11,4) | | | Numerous similar plasmids were also identified in the other strains of *A. baumannii,* *A. pittii, A. haemolyticus* and *A. nosocomialis* |
| II-1b | *mobA/L-repB* | MOB_Q1_ | pALWED3.5  pALWED2.4 | only distant similarity (70-76%). *A.lwoffii* ED2.4 (10,6 kb); *A.cumulans* WCHAc060092 p5_060092 (8,1 kb); *Acinetobacter* sp. WCHAc010034 p7_010034 (10,1 kb); *A. johnsonii* IC001 pIC001A (94,5 kb); *A. venetianus* VE-C3 pAV2 (15,1 kb) | | |  |
| II-1c | *mobA/L-repB* | MOB_Q2_  (MOB_Aci_) | pALWEK1.2  pALWED3.7 | *A.lwoffii* ZS207, pZS-3; (13,8); *Acinetobacter* sp. ACNIH1, pACI-148e; (18,3) | | |  |
| II-1d |  | MOB_Q1_ | - | p3_010060 pXBB1-5 CP026427 pAhaem2126сhe pMMCU1, pAbe229-9 | | | Plasmids from this group were found only in modern strains |
| unique | *repB-parA* | REP_3 (AR3G13) | pALWED1.3 | only distant similarity (76%) and only in relation to the gene for *repB*. *A indicus* CMG3-2, pCMG3-2-2 (16,1); *A.baumannii* 3207, pAba3207a (13,5) | | |  |
| unique | *repB-parA* | REP_3 (AR3G15) | pALWED2.3 | only distant similarity (80% with partial coverage). *A.baumannii* A52, pA52-2 (27,4) | | |  |
| unique | *repB-parA* | ND* | pALWVS1.2 | only distant similarity (от 57-60%) with *A.baumannii* E47, pE47_007 (4,7); *A.haemolyticus* AN54, pAhaemAN54a (4,8); *A.soli* pGFJ5 (5,1 ) and (47-50%) with *Moraxella catarrhalis*, pEMCJH03 (3,5); *Psychrobacter* sp. DAB_AL43B plasmid pP43BP4(6,4). | | | Bears a distant resemblance to pALWEK1.9 |
| Large plasmids | | | | | | | |
| III-1a | *trb*-operon-*parA-parB-tra-*operon | MOB_P_ (P11) | pALWED2.2 | | *A.baumannii* IHIT7853, pIHIT7853-OXA-23 (53,9); *A. pittii* HGSA488, pLS488 (51,3); *A. johnsonii* M19, pFM-M19 (55,0) | | Similar plasmids were detected in strains of other systematic groups (*Klebsiella, Citrobacter*, *E. coli*, *Pseudomonas, Xanthomonas, Stenotrophomonas)* |
| III-1b | *tra-*operon | MOB_Q1_ | - | | *A. wuhouensis* WCHA60 p1_010060 (38,7); *A. lwoffii*: JN49-1 pNDM-BJ01 (47,3) and 32 other plasmids | | Plasmids from this group do not contain described replication genes; most of them carry the *bla*_NDM-1_ gene and were found only in modern bacteria, including a *Providencia* *rettgeri* strain |
| III-3a | *parB-parA-repB* | REP_3 (AR3G4) | pALWED1.2; pALWED2.1 pALWED3.6 pALWVS1.1 pALWEK1.1 | | *A.schindleri* H3, plasmid unnamed1 (223,6); *Acinetobacter s*p. ACNIH1, pACI-df08 (257,7); *A.schindleri* ACE, p1AsACE (17,9); *A. indicus* HY20, pAI01 (11,72); *A. pittii* pAB17H194-1 (88,0); *A.baumannii* D36, pD36-4 (47,5). | |  |
| III-3b | *repB-parA* | REP_3 (AR3G15) | pALWED3.1 | | *A.radioresistence* pARA2 (77,6); *A.radioresistence* pAR1 (88,6); *A.schindleri* pSGAir0122 (156,2); *Acinetobacter sp.* WCHA55, pNDM1 (71,3) | |  |
| III-4a | *rep-parABM-tra-operon* | ND* | pALWED1.1 | | 16 plasmids including *A.baumannii* p34AB (277,9); *A.haemolyticus* pAHTJR1 (306,131); *A.pittii* p2014N21-145-1 (3243,0); *A.johnsonii* pXBB1-9 (398,9) | | the *mobA* gene was not identified |

* ND - Not determined
