# Supplementary material for: Acinetobacter Plasmids: Diversity and Development of Classification Strategies
Source: Front Microbiol. 2020 Nov 13;11:588410. doi: 10.3389/fmicb.2020.588410 (PMC7693717; doi:10.3389/fmicb.2020.588410)
Supplement: Supplementary Table 4 — Small Acinetobacter plasmids belonging to the Group I-2a of MOBQ family. [file Table_4.doc]

**Table S4. Small *Acinetobacter* plasmids belonging to the Group I-2a of MOBQ family**

| Strain | Plasmid, Accession No | Size, kb | mobA identity, % | Accessory genes (proteins) | Source |
| --- | --- | --- | --- | --- | --- |
| *A.lwoffii* ED23-35 | pALWED1.7, CP032116.1 | 4,861 | 100 | LysE translocator | permafrost |
| *A.pseudolwoffii* ED9-5a | pALWED3.3, CP032288.1 | 8,055 | 93,23 | ATP-binding protein; HigA-HigB toxin-antitoxin system | permafrost |
| *A.lwoffii* EK30A | pALWEK1.6, CP032108.1 | 6,886 | 90,43 | LysE translocator; Trypthophan 7-halogenase; type II toxin-antitoxin system | permafrost |
| *A.lwoffii* EK30A | pALWEK1.14 | 4,677 | 93,88 | absent | permafrost |
| *A.lwoffii* VS15 | pALWVS1.5 | 4,677 | 93,88 | absent | permafrost |
| *A.lwoffii* ZS207 | pZS-9, CP019150.1 | 4,348 | 99,46 | absent | Zloty Stok gold mine, Poland |
| *A.lwoffii* ZS207 | pZS-7, CP019148.1 | 5,518 | 96,13 | LysE translocator; IS5 family transposase | Zloty Stok gold mine, Poland |
| *A.lwoffii* ZS207 | pZS-4, CP019147.1 | 6,886 | 90,43 | LysE translocator; Trypthophan 7-halogenase; type II toxin-antitoxin system | Zloty Stok gold mine, Poland |
| *Acinetobacter chinensis*  WCHAc010005 | p3_010005, CP032129.1 | 4,353 | 93,98 | not annotated | sewage, China |
| *A.wuhouensis* WCHA60 | p5_010060, CP031713.1 | 5,424 | 93,55 | type II toxin-antitoxin system | clinical sewage, China |
| *A.baumannii* VB16141 | unnamed2, CP040052.1 | 5,681 | 93,44 | aminoglycoside resisitance(APH(3’)-VIa); BrnT family toxin | blood (Homo sapiens) |
| *A.baumannii* DS002 | pTS4586, CP042206.1 | 4,586 | 93,33 | absent | soil (India) |
| *A.baumannii* A85 | pA85-1b, CP021785.1 | 4,484 | 92,90 | *thyA, folA* | sputum (Homo sapiens) |
| *A.baumannii* Ab14 | pAB14, EF059914.1 | 6,585 | 92,90 | Tn*2007* with *blaOXA-23* | clinic? |
| *A.soli* GFJ2 | pGFJ6, CP016902.1 | 4,066 | 92,90 | not annotated | soil (Thailand) |
| *A.wuhouensis* WCHA62 | p9_010062, CP033129.1 | 4,890 | 92,80 | BrnT family toxin | sewage, China |
| *A.pittii* C54 | pC54_005, CP042369.1 | 4,478 | 94,20 | not annotated | clinical sample (Homo sapiens) |
