# Supplementary material for: Acinetobacter Plasmids: Diversity and Development of Classification Strategies
Source: Front Microbiol. 2020 Nov 13;11:588410. doi: 10.3389/fmicb.2020.588410 (PMC7693717; doi:10.3389/fmicb.2020.588410)
Supplement: Supplementary Table 5 — Distribution of MOBQ plasmids from I-2a group among modern Acinetobacter strains (whole genome shotgun contigs). [file Table_5.doc]

**Table S5. Distribution of MOB_Q_ plasmids from I-2a group among modern *Acinetobacter*** strains (whole genome shotgun contigs)

| Species | Number of  strains  found | Length | Identified accessory genes | Accession No |
| --- | --- | --- | --- | --- |
| *A.pittii* | 9 | 3450 to 5753 | lysine transporter; universal stress protein | MDIK01000004.1; [LLFJ01000058.1](https://www.ncbi.nlm.nih.gov/nuccore/LLFJ01000058); MDIL01000003.1; [NGCD01000031.1](https://www.ncbi.nlm.nih.gov/nuccore/NGCD01000031); [LLHY01001343.1](https://www.ncbi.nlm.nih.gov/nuccore/LLHY01001343); [JRQZ01000130.1](https://www.ncbi.nlm.nih.gov/nuccore/JRQZ01000130); [BBUA01000077.1](https://www.ncbi.nlm.nih.gov/nuccore/BBUA01000077); [MDIA01000002.1](https://www.ncbi.nlm.nih.gov/nuccore/MDIA01000002); [MDIC01000003.1](https://www.ncbi.nlm.nih.gov/nuccore/MDIC01000003) |
| *A.lwoffii* | 4 | 4863 to 8359 | LysE family translocator | ACPN01000207.1; [APQS01000028.1](https://www.ncbi.nlm.nih.gov/nuccore/APQS01000028);   | [VCND01000094.1](https://www.ncbi.nlm.nih.gov/nuccore/VCND01000094); | | --- |   [WURQ01000039.1](https://www.ncbi.nlm.nih.gov/nuccore/WURQ01000039) |
| *A.bereziniae* | 4 | 3718 to 5575 | not identified | [BKPS01000185.1](https://www.ncbi.nlm.nih.gov/nuccore/BKPS01000185); [CDEL01000176.1](https://www.ncbi.nlm.nih.gov/nuccore/CDEL01000176); [BBLJ01000094.1](https://www.ncbi.nlm.nih.gov/nuccore/BBLJ01000094); [AIEI01000192.1](https://www.ncbi.nlm.nih.gov/nuccore/AIEI01000192) |
| *A.gerneri* | 4 | 3636 to 6948 | ATPase involved in DNA repair; inorganic pyrophosphatase | [APPN01000035.1](https://www.ncbi.nlm.nih.gov/nuccore/APPN01000035);  [BBLI01000098.1](https://www.ncbi.nlm.nih.gov/nuccore/BBLI01000098);  [ASYY01000024.1](https://www.ncbi.nlm.nih.gov/nuccore/ASYY01000024);  [APPN01000001.1](https://www.ncbi.nlm.nih.gov/nuccore/APPN01000001) |
| *A.junii* | 3 | 5615 to 5805 | not identified | [BBSD01000143.1](https://www.ncbi.nlm.nih.gov/nuccore/BBSD01000143); ASYZ01000001.1; APPS01000003.1 |
| *A.johnsonii* | 1 | 6,082 | cation transporter; isoprenylcysteine carboxylmethyltransferase family protein | RHXI01000085.1 |
| *A.haemolyticus* | 2 | 5042 to 6204 | BrnT family toxin; helix-turn-helix domain-containing protein | [WTTI01000150.1](https://www.ncbi.nlm.nih.gov/nuccore/WTTI01000150); [WTTS01000135.1](https://www.ncbi.nlm.nih.gov/nuccore/WTTI01000150) |
| *A.schindleri* | 2 | 4958 to 5850 | SulP family inorganic anion transporter;  universal stress protein | [APPI01000006.1](https://www.ncbi.nlm.nih.gov/nuccore/APPI01000006); [QCWW01000075.1](https://www.ncbi.nlm.nih.gov/nuccore/QCWW01000075) |
| *A.indicus* | 1 | 5064 | not identified | PRDY01000063.1 |
| *A.soli* | 1 | 4556 | not identified | [BKCR01000045.1](https://www.ncbi.nlm.nih.gov/nuccore/BKCR01000045) |
| *A. idrijaensis* | 1 | 3358 | not identified | [JQCU01000143.1](https://www.ncbi.nlm.nih.gov/nuccore/JQCU01000143) |
